# Extraction of needs based on perceived stigma and their alignment with NANDA in people living with HIV in Iran

**DOI:** 10.1186/s12912-026-04458-6

**Published:** 2026-03-25

**Authors:** Hamideh Ebrahimi, Shima Sadat Aghahosseini

**Affiliations:** Lahore School of Nursing, The University of Lahore, Lahore, Pakistan

**Keywords:** Stigma, Nursing diagnosis, AIDS/HIV, NANDA, Qualitative study

## Abstract

**Background:**

AIDS/HIV is a major public health challenge that, in addition to physical complications, is accompanied by social stigma and negative impacts on mental health and quality of life. Identifying the needs of people living with HIV (PLHIV) and aligning them with NANDA nursing diagnoses can strengthen person-centered and evidence-based care. This study aimed to extract needs based on perceived stigma and map them to NANDA nursing diagnosis patterns.

**Materials and methods:**

This study employed a directed qualitative content analysis approach, based on the framework proposed by Hsieh and Shannon (2005). It was conducted in 2024 with 21 people living with HIV (PLHIV) recruited from health centers in East Tehran, Iran. Data were collected through semi-structured interviews using purposive sampling, and analysis was guided by six predefined NANDA nursing diagnosis domains.

**Results:**

Most participants were male, with a mean age of 38.52 ± 9.75 years. Directed content analysis resulted in the identification of 11 main categories and 42 subcategories. Findings indicated that perceived stigma influences the multidimensional needs of PLHIV, including physical, psychological, social, sexual, coping-related, and spiritual needs within NANDA diagnosis patterns.

**Conclusion:**

Perceived stigma broadly affects the physical, psychological, social, sexual, and spiritual care needs of PLHIV. The use of NANDA nursing diagnosis patterns enables a systematic explanation of these needs and guides the development of comprehensive, individualized, culturally-congruent, and evidence-informed nursing care, which may improve treatment adherence and enhance quality of life.

**Clinical trial number:**

Not applicable.

**Supplementary Information:**

The online version contains supplementary material available at 10.1186/s12912-026-04458-6.

## Background

HIV/AIDS is recognized as one of the most critical public health crises, leading to immune deficiency symptoms, facilitating the invasion of opportunistic infections, and ultimately resulting in vital organ involvement and death [1]. This disease is ranked as the second most significant infection-related cause of death worldwide [2]. The total number of people living with HIV in Iran in 2024 is estimated at approximately 44,105 individuals [3]. According to reported statistics, the prevalence of HIV in Iran shows an upward trend, while many developing countries have been able to control the disease to some extent [4].

Despite the efforts of health centers to control the disease, HIV-related stigma remains a major barrier to case identification and patient care and is considered a global phenomenon across different cultures [5, 6]. This stigma can disrupt the beliefs and daily functioning of people living with HIV [7], and may lead to reduced self-esteem, feelings of guilt, maladaptive behaviors, physical complaints such as sleep disturbance and chronic fatigue, and ultimately a decline in quality of life [8]. Stigma associated with AIDS is one of the key challenges in long-term care [9]. Studies indicate that negative attitudes of nurses toward people living with HIV (PLHIV), driven by fear of infection or lack of knowledge, reduce the quality of care [10].

In this context, the use of standardized nursing language particularly NANDA nursing diagnoses as a scientific framework for identifying human responses to disease and treatment is of great importance. Perceived stigma, as a psychosocial phenomenon, represents a human response to social attitudes and discrimination that can negatively affect emotional, behavioral, and social functioning. In nursing, these human responses can be systematically identified and categorized using standardized languages such as NANDA nursing diagnoses. For example, feelings of isolation, anxiety, or ineffective coping related to stigma can be translated into specific NANDA diagnoses, enabling nurses to design targeted, evidence-based interventions that address both psychosocial and physical needs. Thus, perceived stigma provides a conceptual link between patients’ lived experiences and structured nursing assessments, highlighting the importance of integrating qualitative insights into standardized nursing care. NANDA nursing diagnoses enable the systematic identification of patients’ physical, psychological, social, and spiritual needs and contribute to the design of evidence-based and person-centered nursing care [11].

Qualitative studies have documented a wide range of psychosocial challenges among people living with HIV/AIDS, including fear, anxiety, depression, social stigma, social isolation, and disruptions in family relationships, all of which influence health management and treatment adherence [12]. The lived nature of these experiences particularly social stigma and the complex psychosocial needs of individuals living with HIV requires in-depth, interpretive data grounded in participants’ personal meanings and contexts. In this regard, quantitative approaches that primarily rely on clinical or numerical data are insufficient to adequately capture these subjective, contextual, and socially embedded dimensions. Nevertheless, existing qualitative findings are often reported in a largely descriptive manner and remain insufficiently integrated into structured nursing knowledge. Therefore, there is a need for an analytic approach that systematically organizes qualitative evidence within established theoretical frameworks to facilitate the development of standardized nursing diagnoses. Directed content analysis, as a qualitative methodological approach, allows existing theory to guide the interpretation of qualitative data and offers a particularly suitable strategy for examining patients’ experiences within cultural and social contexts, where beliefs, values, and family structures play a critical role in shaping responses to illness.

Previous studies on HIV/AIDS have mostly been quantitative, focusing on clinical indicators and general outcomes, while existing qualitative studies have primarily provided descriptive insights into the psychosocial experiences of patients. However, these findings have not been systematically organized within standardized nursing language, particularly NANDA nursing diagnoses, and do not comprehensively reflect patients’ needs based on perceived stigma. The present study addresses this gap by using directed content analysis and the NANDA framework to translate the lived experiences of people living with HIV/AIDS into standardized nursing diagnoses, providing culturally grounded evidence from Iran.

Accordingly, the present study was conducted in Iran to align the needs of people living with HIV/AIDS, based on perceived stigma, with NANDA nursing diagnosis patterns. Religious beliefs, social norms, and HIV-related taboos in Iran contribute to shame, fear of disclosure, and stigma, which negatively affect psychological well-being and treatment adherence. Family support and social acceptance are crucial for coping.

By translating these culturally and socially embedded experiences into standardized NANDA nursing diagnoses, the findings of this study have direct practical implications for nursing practice. First, they can enhance the accuracy and comprehensiveness of the nursing diagnosis process, enabling nurses to systematically identify psychosocial, emotional, and social needs that are often overlooked. Second, they provide a framework for developing targeted, evidence-based interventions that address stigma, promote coping strategies, and improve psychological well-being. Third, integrating patients’ lived experiences into nursing care supports person-centered approaches, ensuring that care plans are culturally sensitive and aligned with patients’ values and social contexts. Ultimately, this approach has the potential to reduce HIV-related stigma in healthcare settings, improve adherence to treatment, and elevate overall quality of care, highlighting the crucial role of culturally informed nursing assessments in addressing complex psychosocial challenges.

## Methods

This qualitative study was conducted using a directed content analysis approach based on the model proposed by Hsieh and Shannon (2005), with the aim of extracting needs related to perceived stigma and mapping them onto NANDA-I nursing diagnosis patterns [1]. The study was conducted in 2024 with 21 people living with HIV (PLHIV) recruited from the East Behavioral Health Center in Tehran, Iran. All NANDA-I nursing diagnosis domains were initially considered as the theoretical framework for data analysis. During the analysis process, experiences related to perceived stigma were found to be meaningfully identifiable within six domains: Health Perception–Health Management, Self-Perception–Self-Concept, Role–Relationship, Sexuality–Reproductive, Coping–Stress Tolerance, and Value–Belief. Accordingly, these six domains were used as the final conceptual framework for analysis.

A total of 21 participants were selected using purposive sampling with maximum variation. Sampling continued until data saturation was achieved. Inclusion criteria included: age ≥ 18 years; confirmed diagnosis of HIV based on medical records and physician confirmation; representation of different disease stages; inclusion of both female and male participants; Iranian nationality; ability to speak and understand Persian; adequate physical and psychological capacity to participate in interviews; and willingness to take part in the study. Participants were recruited from health centers affiliated with Shahid Beheshti University of Medical Sciences. Initial access was facilitated by center staff, who introduced the study to potential participants. Individuals who expressed interest were contacted by the first author and provided with detailed information about the study before scheduling interviews.

Data were collected through in-depth semi-structured interviews conducted by the first author, a PhD candidate in Nursing with formal training in qualitative research methods. The first author had no prior therapeutic, educational, or professional relationship with participants, minimizing potential bias. Each participant took part in one interview session lasting approximately 45–60 min. All interviews were audio-recorded with participants’ informed consent and transcribed verbatim by the first author. No follow-up interviews or observational field notes were conducted. An interview guide was used to facilitate interviews and focused on experiences of perceived stigma, its consequences, and related care needs. The full interview guide is provided in Supplementary File 1. Key questions included: “Can you describe any experiences of stigma you have encountered due to your HIV status?” “How have these experiences affected your daily life and care needs?” and “What kind of support or interventions do you feel would help you manage these challenges?” Interviews began with general open-ended questions to establish rapport and were followed by probing questions to clarify and expand participants’ responses.

Data were analyzed using a directed content analysis approach based on Hsieh and Shannon (2005). Six predefined NANDA-I domains provided the initial deductive coding framework. Interview transcripts were reviewed repeatedly and analyzed line by line to identify meaning units, which were condensed and assigned initial codes. Each code was systematically compared with the operational definitions of NANDA-I domains and diagnoses. During the initial stage of analysis, all NANDA-I domains were considered as potential analytical categories. Domains were selected for the final framework based on three criteria: [1] frequency and richness of relevant data [2], conceptual consistency between participants’ experiences and NANDA-I definitions, and [3] the possibility of translating findings into standardized nursing diagnoses. As a result, six domains showed substantial conceptual alignment with stigma-related experiences and were retained. Data segments were assigned to domains according to their dominant meaning. In cases where data reflected multiple dimensions, classification was guided by the primary focus of the participant’s experience. When data did not fit predefined categories, additional codes were generated inductively using a conventional approach. The first author performed the initial coding, and all codes and categories were reviewed by the second author. Discrepancies were resolved through discussion and consensus, ensuring analytical rigor and consistency. This iterative process integrated both theory-driven and data-driven insights.

Given the sensitive nature of HIV research, the researchers maintained awareness of their own roles, assumptions, and potential biases throughout the study. The first author acknowledged that her professional background and qualitative research training could influence data collection and interpretation. To minimize these effects, reflexive journaling was practiced, and regular discussions were held within the research team to reflect on how personal perspectives might shape interactions and analysis. Several measures were implemented to ensure participants’ comfort and safety. Interviews were conducted in private rooms within health centers, with only the participant and researcher present. Participants were informed of their right to decline any question or withdraw at any time without consequences. All data were anonymized, and identifying information was removed from transcripts. Audio recordings and transcripts were stored on password-protected devices accessible only to the research team. A neutral and nonjudgmental approach was maintained throughout interviews to minimize stress and discomfort. To ensure rigor and trustworthiness, the criteria proposed by Guba and Lincoln [14], including credibility, dependability, confirmability, and transferability, were applied. Member checking was conducted with all participants after preliminary analysis, and peer debriefing sessions were held with experienced qualitative researchers not involved in data collection. The first author conducted the primary analysis, and the second author reviewed and validated codes and interpretations. Reflexive discussions were held throughout the research process to minimize potential bias.

The study was approved by the Research Ethics Committee of Shahid Beheshti University of Medical Sciences. All procedures complied with the Declaration of Helsinki, and written informed consent was obtained from all participants.

## Results

In this study, 21 individuals living with HIV participated. The majority of participants were male (76.2%). The mean age of participants was 38.52 years (SD = 9.75). Regarding educational level, the highest frequency belonged to participants with a high school diploma (38.1%), followed by middle school education (33.3%). The mean duration of HIV infection was 5.1 years (SD = 2.5).

In terms of marital status, 47.6% of participants were single, 28.6% were married, 14.3% were divorced, and 9.5% were widowed. Participants demonstrated acceptable diversity in age, duration of infection, and socioeconomic status, supporting maximum variation in sampling. Detailed demographic characteristics of participants are presented in Table 1.

**Table 1:** Demographic characteristics of participants

Variable	Group	*n* (%) / Mean ± SD
Gender	Male	16 (76.2%)
	Female	5 (23.8%)
Education level	Middle school	7 (33.3%)
	High school diploma	8 (38.1%)
	Associate degree	1 (4.8%)
	Bachelor’s degree	4 (19.0%)
	Master’s degree	1 (4.8%)
Marital status	Single	10 (47.6%)
	Married	6 (28.6%)
	Divorced	3 (14.3%)
	Widowed	2 (9.5%)
Duration of HIV infection (years)	5.10 ± 5.26	
Age (years)	38.52 ± 9.75	

Based on directed content analysis within the framework of predefined NANDA nursing diagnosis patterns, a total of six patterns, 11 main categories, and 42 subcategories were identified. An example of the process of extracting categories and subcategories up to the initial coding stage is presented in Table 2, and representative examples with participant quotations are shown in Table 3. All extracted data related to perceived stigma were meaningfully classified within the predefined NANDA-I domains and categories, and no data remained outside this framework; therefore, no new categories were generated, the complete list of all extracted categories and subcategories is provided in Table 2, and along with participant quotations, representative examples are presented in Table 3.

**Table 2:** Categories and Subcategories Extracted Based on Directed Content Analysis and NANDA-I Nursing Diagnosis Patterns

NANDA-I	NANDA-I Nursing Diagnoses	Category	Subcategory
Self-Perception–Self-Concept	Fear; Anxiety; Anticipatory Anxiety; Death Anxiety; Depression; Risk for Loneliness; Hopelessness; Low Self-Esteem; Risk for Impaired Human Dignity; Risk for Self-Harm	Negative Emotions	• Feeling of Depression • Sense of Inferiority and Low Self-Worth • Isolation and Loneliness • Inner Turmoil • Doubt and Confusion • Shock and Helplessness • Anger and Desire for Revenge • Impaired Self-Esteem • Fear of Death
Role–Relationship	Social Isolation; Impaired Social Interaction; Grieving; Anticipatory Grieving; Dysfunctional Family Processes; Risk for Other-Directed Violence	Suffering from Social Stigma	• Inappropriate Family and Social • Interactions Blame and Accusation by Others • Discriminatory Behaviors of Healthcare Providers • Manifestation of Disturbing Fear in Others • Labeling and Misjudgment by Others • Fear-Inducing Interactions • Employment and Occupational Problems
Health Perception–Health Management	Health-Risk Behavior; Ineffective Health Management; Risk for Ineffective Therapeutic Regimen Management; Risk for Nonadherence; Risk for Injury; Risk for Infection	Disruption of Normal Life	• Concealment of Illness • Loss of Self-Efficacy • Health-Damaging Behaviors
Coping–Stress Tolerance	Ineffective Coping; Impaired Individual Resilience; Post-Trauma Syndrome; Deficient Support System; Risk for Suicide; Compromised Family Coping; Ineffective Community Coping	Denial and Avoidance	• Refusal to Accept Illness • Denial of Disease • Conscious Forgetting • Emotional Suppression and Concealment Excessive • Use of Sedative Medications
		Confronting Disease Complications	• Physical Symptoms • Neuropsychiatric Disorders
		Health and Treatment Problems	• Insurance Coverage Problems • Medication Supply Problems • Treatment Difficulties
		Positive Coping with Illness	• Breaking the Silence and Talking About the Disease • Voluntary Medical Consultation
Sexuality–Reproductive	Ineffective Sexuality Pattern; Impaired Spousal Role Performance; Risk for Impaired Maternal–Fetal Attachment	Instability in Marital Foundation	• Fear of Rejection • Fear of Separation and Divorce • Emotional Divorce
		Parenting Challenges	• Concerns and Confusion About • Parenthood Worries About Pregnancy Outcomes
		Challenges in Managing Sexual Relationships	• Decreased Sexual Self-Confidence • Reduced Quantity and Quality of Sexual Relations
Value–Belief	Moral Distress; Spiritual Distress; Readiness for Enhanced Religiosity	Meaning-Making of Life	• Perceiving Illness as Divine • Punishment or Will Comfort Through Prayer and Seeking Healing • Transition from Material to Spiritual Values • Self-Focus Rather Than Others

**Table 3:** Sample of the process of extracting categories and subcategories with participant quotations

NANDA Nursing Diagnosis Pattern	Category	Subcategory	Initial Code	Participant Quotation
Health Perception–Health Management Pattern	Disruption of normal life	Compulsion to hide the disease	Treatment concealment, non-attendance	“I used to hide my medications and did not go to the health center for two months after learning about my diagnosis.”
Self-Perception–Self-Concept Pattern	Negative emotions	Feelings of depression and hopelessness	Low self-esteem, hopelessness	“I felt worthless and humiliated; I wanted to die.”
Role–Relationship Pattern	Suffering from social stigma	Manifestation of fear and harm from others	Experience of social discrimination	“When I went to a party, I saw hands being washed and children wiped with alcohol.”
Sexuality–Reproductive Pattern	Challenges in managing sexual relationships	Decreased quantity and quality of sexual relations	Reduced marital intimacy	“This disease affected my relationship with my spouse and made it cold.”
Coping–Stress Tolerance Pattern	Denial and avoidance	Disease denial	Disease denial, ineffective coping	“I just couldn’t accept that I had AIDS and no longer listened to anyone.”
Value–Belief Pattern	Meaning-making in life	Calmness through spiritual practice	Spiritual reliance	“I go on pilgrimages, and it helps me feel calmer.”

### Health perception–health management pattern

According to the NANDA framework, the Health Perception–Health Management pattern refers to individuals’ perceptions of their health status and their behaviors related to maintaining and promoting health. In the present study, directed content analysis led to the identification of two main categories and eight subcategories within this pattern [2]. Participants’ lived experiences, such as concealing their illness, irregular attendance at healthcare centers, discontinuation of healthy behaviors, and delays in seeking medical care, indicated disruptions in this domain and reflected weakened self-care and ineffective health management resulting from perceived stigma.

Most participants (*n* = 20) reported experiencing “Disruption of Normal Life,” which included three subcategories: compulsion to hide the disease, loss of self-efficacy, and health-lowering behaviors. These experiences were manifested through treatment concealment, discontinuation of healthy behaviors, and delays in seeking healthcare, reflecting weakened self-management and fear of social exposure.

**Representative quotations included**:

*I used to hide my medications* (P13, Female, single).

*I stopped exercising and started smoking constantly* (P6, Male, single).

*I developed binge eating because my mind was constantly occupied by others’ behaviors* (P9, Female, divorced).

One participant stated: “*I used to hide my medications. At first*,* to avoid thinking about the disease bothering me*,* I would take diazepam at night.”* (P13, Female, single).

Several participants (*n* = 12) described “Denial and Avoidance,” which included five subcategories: non-acceptance of the disease, denial, deliberate forgetting, suppression of emotions, and excessive use of sedatives. These behaviors indicated ineffective coping strategies and emotional withdrawal in response to stigma.

**Representative quotations included**:

*For two months after my diagnosis*,* I still could not accept it *(P20, Female, widowed).

*I couldn’t believe that I had HIV *(P11, Male, married).

*I ignored everyone and did things my own way *(P15, Male, married).

**One participant reported**:

*Even two months after learning about my diagnosis*,* I still could not accept it and did not go to the health center* (P20, Female, widowed).

Overall, this pattern demonstrated that perceived stigma weakens health management, fosters risky behaviors, and reduces treatment adherence among people living with HIV.

### Self-perception–self-concept pattern

Within the NANDA framework, the Self-Perception–Self-Concept pattern refers to individuals’ attitudes, feelings, and perceptions about themselves, including self-esteem, body image, and personal identity [2]. In this study, directed content analysis identified one main category and nine subcategories within this pattern. Participants’ experiences, such as feelings of worthlessness, hopelessness, anxiety, depression, and disturbances in personal identity, indicated impairments in this psychological domain and collectively reflected weakened self-concept associated with perceived social stigma.

Most participants (*n* = 13–15) reported experiencing intense negative emotions and impaired self-concept, including fear, anxiety, depression, hopelessness, feelings of worthlessness, low self-esteem, and disturbances in body image and personal identity. In some cases, these experiences were accompanied by self-harming thoughts and behaviors, reflecting severe psychological distress related to stigma and social marginalization.

**Representative quotations included**:

*I had turned AIDS into a giant for myself *(P4, Male, married).

*I felt worthless and humiliated *(P7, Male, married).

*My life felt meaningless*,* and I wanted to die *(P12, Female, married).

One participant stated: “I had turned AIDS into a giant for myself. I felt humiliated and worthless. I wanted to die.” (P4, Male, married).

In contrast, several participants (*n* = 6) demonstrated adaptive capacities, such as readiness to regain hope and improve self-efficacy, indicating the potential effectiveness of supportive and self-concept-enhancing nursing interventions in strengthening psychological resilience.

**Representative quotations included**:

*I try to stay hopeful and take care of myself *(P9, Female, divorced).

*I learned how to manage my condition better *(P14, Male, single).

One participant reported: *“Whenever I need to talk to the doctor*,* I go to the center and show them my test results”* (P10, Male, single).

### Role–relationship pattern

According to the NANDA framework, the Role–Relationship pattern focuses on the quality of individuals’ relationships with family members, spouses, friends, and the community, as well as the performance of social and familial roles [3]. In this study, directed content analysis identified two main categories and eight subcategories within this pattern. Participants’ lived experiences demonstrated that perceived stigma disrupted interpersonal relationships, weakened social support, and impaired spousal and parental role functioning.

Nearly half of the participants (*n* = 10) reported experiencing “Suffering from Social Stigma,” which included inappropriate family and social interactions, rejection, blame, discriminatory behaviors, labeling, and impaired social interaction. These experiences undermined participants’ social status and weakened their roles within the family and community.

**Representative quotations included**:

*My value and respect disappeared in my spouse’s family* (P11, Male, married).

*When I went to a party*,* they washed their hands and used alcohol on their children *(P9, Female, divorced).

*They look at me as if I am a dangerous person* (P6, Male, single).

**One participant stated**:

*My value and respect disappeared in my spouse’s family after my disease became known *(P11, Male, married).

Concerns of HIV-positive parents about their children’s future and societal reactions further disrupted family processes and contributed to ineffective role performance, particularly in parental and spousal roles.

Several participants (*n* = 7) demonstrated “Readiness for Improved Communication,” highlighting the supportive role of counseling centers and family education in improving interpersonal relationships.

Representative quotations included:

*The center teaches our families how to communicate with us* (P8, Male, divorced).

*Counseling helped my family understand me better *(P13, Female, single).

In some narratives, emotional responses to social humiliation were associated with anger and aggressive reactions toward others.

### Sexuality–reproductive pattern

In the NANDA framework, the Sexuality–Reproductive pattern refers to individuals’ attitudes, emotions, and behaviors related to sexual relationships, intimacy, reproduction, and childbearing [2]. In this study, directed content analysis identified three main categories and seven subcategories within this pattern. Participants’ experiences, including reduced marital intimacy, fear of rejection, feelings of guilt, and concerns about transmitting the disease to partners or children, indicated disruptions in this domain. The identified subcategories reflected the combined influence of perceived stigma and cultural taboos on sexual functioning and reproductive decision-making.

Due to the cultural sensitivity and taboo surrounding discussions of sexuality, only a limited number of participants (*n* = 4–6) were willing to openly report experiences related to sexual and reproductive concerns. Nevertheless, those who did share their experiences provided rich insights into the challenges faced in this domain.

Several participants (*n* = 5) reported “Instability in Marital Foundations,” including fear of rejection, fear of separation and divorce, and emotional divorce, which resulted in reduced intimacy and emotionally distant marital relationships.

**Representative quotations included**:

*This disease has cooled my relationship with my spouse *(P15, Male, married).

*After my diagnosis*,* our emotional connection became very weak *(P8, Male, divorced).

*I’m always afraid that my partner may leave me* (P11, Male, married).

*During pregnancy*,* I was constantly worried that my baby might be infected* (P12, Female, married).

A few participants (*n* = 4) described “Parenting Challenges,” expressing concerns and confusion related to parenthood and pregnancy outcomes.

**Representative quotations included**:

*During pregnancy*,* I was worried that my baby might also be infected (P12*,* Female*,* married)*.

*I was constantly anxious about my child’s future and how others might treat them (P21*,* Female*,* married*,* mother *

Several participants (*n* = 6) reported “Challenges in Managing Sexual Relationships,” reflecting reduced sexual self-confidence, feelings of guilt, and decreased frequency and quality of sexual relations, sometimes accompanied by self-blame.

**Representative quotations included**:

*Because my spouse was also infected*,* I blame myself *(P16, Male, divorced).

*My self-confidence in sexual relationships decreased a lot *(P9, Female, divorced).

*I feel guilty whenever I think about intimacy *(P3, Male, single).

**One participant stated**:

*Because my spouse was also infected*,* I blame myself and my self-confidence has decreased a lot* (P16, Male, divorced).

### Coping–stress tolerance pattern

Within the NANDA framework, the Coping–Stress Tolerance pattern refers to individuals’ methods of dealing with psychological, social, and physical stressors. In this study, directed content analysis identified four main categories and nine subcategories within this pattern [3]. The findings demonstrated that participants employed diverse strategies, such as denial, avoidance, emotional suppression, and, conversely, seeking social support and open communication. The subcategories represented a continuum of maladaptive to adaptive coping strategies developed in response to perceived stigma and psychosocial stressors.

Several participants (*n* = 11) reported “Denial and Avoidance,” including disease denial, deliberate forgetting, emotional suppression, and impaired personal flexibility, indicating that some participants resorted to ineffective coping strategies to reduce psychological stress.

**Representative quotations included**:

*I just couldn’t accept that I had AIDS *(P19, Male, single).

*I ignored everyone and did things my own way* (P15, Male, married).

*At first*,* I pretended nothing had happened* (P7, Male, married).

**One participant stated**:

*I just couldn’t accept that I had AIDS. I no longer listened to anyone and did things my way* (P19, Male, single).

Some participants (*n* = 8) reported “Confronting Disease Complications,” including physical and neuropsychological symptoms that reduced individuals’ capacity to manage their condition.

**Representative quotations included**:

*Because of nerve problems*,* I couldn’t work properly anymore* (P6, Male, single).

*My physical weakness made everything harder* (P11, Male, married).

“Several participants (*n* = 9) reported “Healthcare and Treatment Challenges,” referring to structural limitations such as inadequate insurance coverage, difficulty accessing medications, and limited healthcare access, which weakened family and social coping.

**Representative quotations included**:

*Insurance doesn’t cover most of my tests *(P3, Male, single).

*Many hospitals don’t accept us for surgery *(P14, Male, single).

Some participants (*n* = 7) reported “Positive Coping with Disease,” reflecting efforts to use adaptive strategies such as breaking silence, discussing the disease, and voluntarily seeking support and care.

**Representative quotations included**:

*I talk openly about my illness now* (P9, Female, divorced).

*Getting support helped me continue treatment *(P5, Male, widowed).

*I learned how to take better care of myself *(P17, Male, single).

In this pattern, some participants (*n* = 7) actively used adaptive coping strategies to confront and challenge HIV-related stigma. By openly discussing their condition, seeking professional and social support, and engaging in self-care behaviors, they attempted to reduce the negative impact of stigma on their lives. These participants demonstrated resilience and agency, using constructive coping mechanisms to overcome stigma-related psychological and social difficulties.

Some narratives also indicated intensified psychological pressure (e.g., isolation, depression, and risk of suicide) under conditions of insufficient formal and informal support.

**One participant noted**:

*Isolation and depression can lead to suicide. On the other hand*,* insurance coverage is completely insufficient for tests and treatments* (P3, Male, single).

### Value–belief pattern

According to the NANDA framework, the Value–Belief pattern refers to individuals’ religious, spiritual, and personal values that influence how illness experiences are interpret [4]. In this study, directed content analysis identified one main category and four subcategories within this pattern. Some participants used religious beliefs, prayer, and spiritual practices to interpret the disease as an opportunity for spiritual growth and closeness to God. These experiences facilitated life meaning-making and redefinition of the illness experience. The subcategories within this pattern highlighted the role of spiritual and value systems in enhancing psychological resilience in the context of perceived stigma.

Several participants with strong religious beliefs (*n* = 6–8) repeatedly referred to this pattern, interpreting the disease not only as a threat but, in some cases, as an opportunity for spiritual growth, personal development, and closeness to God. Religious beliefs, prayer, and spiritual practices contributed to psychological comfort, enhanced coping, and readiness to improve religiosity.

**Representative quotations included**:

*My grandmother said this disease helps cleanse your sins and brings you closer to God* (P7, Male, married).

*Praying gives me peace when I feel hopeless* (P12, Female, married).

*My faith helps me tolerate this condition *(P5, Male, widowed).

Some participants also reported that engaging in spiritual practices, such as pilgrimage and prayer, helped them cope with the disease and experience greater peace.

**Representative quotations included**:

*I feel calmer when I go on pilgrimages *(P17, Male, single).

*Visiting holy places gives me strength* (P9, Female, divorced).

**One participant expressed**:

*A patient must have strong faith; I feel calmer when I go on pilgrimages *(P17, Male, single).

## Discussion

In this study, NANDA-I diagnostic patterns were used as the theoretical framework for data analysis in order to translate qualitative data derived from experiences of perceived stigma among people living with HIV into standardized nursing language. By focusing on human responses to illness and treatment, this framework enables the formulation of patients’ physical, psychological, social, sexual, and spiritual needs into actionable nursing diagnoses and provides a foundation for designing person-centered care plans [1, 5]. Accordingly, the findings of this study go beyond merely describing lived experiences and, through classification within NANDA patterns, offer a practical pathway for care planning. In this way, nurses can identify potential diagnoses for each pattern, underlying factors such as fear of stigma and disclosure, and expected outcomes such as reduced treatment adherence, and subsequently design targeted interventions based on these assessments [6, 7]. The findings also demonstrated that perceived stigma among people living with HIV in Iran generates or intensifies multidimensional care needs that can be interpreted within the framework of NANDA diagnostic patterns and translated into practical care plans. Although previous studies have reported associations between stigma, psychological consequences, and reduced quality of life [4–6], in the Iranian socio-cultural context, these experiences are shaped by cultural taboos, moral shame, fear of social judgment, and the prominent role of family and religion. These contextual factors influence pathways of adaptation, disclosure or concealment of illness, and interaction with the healthcare system, thereby distinguishing patients’ experiences from those reported in some other societies [8–10]. In the following sections, each NANDA-I pattern is discussed in relation to relevant literature and interpreted within the Iranian socio-cultural context.

### Health perception–health management pattern

In this pattern, disruptions in daily routines and patterns of denial and avoidance indicated that perceived stigma directs disease management behaviors toward treatment concealment, delayed healthcare seeking, and the use of ineffective coping strategies. This finding is consistent with existing evidence showing that fear of stigma can lead to delays in accessing healthcare services and reduced treatment adherence [6, 11]. In Iran, due to the social sensitivity surrounding HIV and concerns about disclosure within the family, place of residence, and workplace, treatment concealment becomes a strategy for preserving social identity [12, 13].

From a nursing perspective, these findings correspond to diagnoses such as *Ineffective Health Management* and *No adherence to Treatment*. Under these circumstances, nurses can design effective care plans by assessing disclosure safety, providing targeted self-care education, enhancing treatment literacy, and linking patients to support groups [10, 14]. Evidence from care guideline studies conducted in Iran also indicates that education, cultural awareness, and preparation of stigma-sensitive care environments are prerequisites for the effectiveness of these interventions [10, 15].

### Self-perception–self-concept pattern

A substantial proportion of the data indicated that perceived stigma was associated with depression, hopelessness, feelings of worthlessness, and self-harming thoughts, which have also been reported in the literature as major consequences of stigma [16, 17]. From a cultural perspective, HIV-related stigma in Iran is often linked to moral interpretations and social labeling, such that individuals experience not only illness but also social judgment, resulting in damage to their self-concept [18].

These findings correspond to diagnoses such as *Low Self-Esteem*, *Hopelessness*, and *Risk for Suicide*. Nursing interventions, including depression screening, supportive counseling, and family education aimed at reducing blame, can contribute to rebuilding hope and enhancing self-efficacy [19, 20]. This approach is consistent with multilevel stigma-reduction strategies.

### Role–relationship pattern

The findings showed that perceived stigma leads to rejection, blame, discrimination, and impaired social interactions, thereby weakening spousal and parental roles. In Iranian society, where family plays a central role in providing support or rejection, disclosure of HIV status may result in decreased social status, reduced respect within the spouse’s family, and changes in family relationship patterns [21–23]. This issue is not limited to the family level; experiences of stigma within the healthcare system may also lead to avoidance of services, concealment in interactions with healthcare providers, and reduced motivation for health maintenance [23].

Therefore, in the Iranian context, “relationships” are simultaneously affected at two levels: [1] the family–social network and [2] the therapeutic relationship with the healthcare system. Both levels may weaken treatment adherence [10].

From a nursing perspective, this pattern aligns with diagnoses such as *Impaired Social Interaction* and *Interrupted Family Processes*. Accordingly, care should not rely solely on individual recommendations but must be designed using family-centered and network-based approaches. Stigma-reduction guidelines emphasize that identifying individual and family needs should be conducted through structured interviews, observation, open-ended questions, and family-focused sessions, particularly for more vulnerable groups (e.g., young, single, socially isolated individuals, or those infected by their partners) [15, 17, 24].

Within this framework, nurses can facilitate family education sessions using simple, nonjudgmental language to shift family attitudes from blame to support and maintain patients’ daily interactions. At the same time, facilitating connections to peer and support groups can strengthen a sense of belonging and social support and help prevent social isolation.

### Sexuality–reproductive pattern

In this pattern, stigma and fear of transmission destabilized marital relationships and challenged decision-making regarding pregnancy and childbearing. This finding is consistent with existing evidence on the impact of stigma on intimate relationships and reproductive decisions [25, 26]. In Iran, cultural taboos surrounding discussions of sexuality limit the expression of educational and counseling needs, which may manifest as emotional distance or hidden conflicts within relationships [11]. These taboos are often more restrictive for women, who may experience greater social control, fear of judgment, and barriers to openly discussing sexual and reproductive concerns [27]. As a result, women may be less willing to disclose such experiences or participate in related research, which may partly explain their lower representation in the present sample.

These findings correspond to diagnoses such as *Ineffective Sexuality Pattern* and *Anxiety Related to Disease Transmission*. Providing confidential sexual counseling, education on prevention of mother-to-child transmission, and couple-centered support may improve relationships and reduce anxiety.

### Coping–stress tolerance pattern

The findings indicated that perceived stigma led some patients to ineffective coping strategies (denial, emotional suppression, avoidance of care and treatment), while in others it activated adaptive coping pathways (seeking support, acceptance, and efforts to maintain care continuity).The study by Bauermeister et al. supports this pattern, showing that exposure to stigma within supportive peer or dialogue-based environments particularly those emphasizing experience sharing and reducing loneliness may be associated with reduced perceived stigma. In contrast, when discussions focus mainly on fear and anticipated stigma, psychological distress and perceived stigma may increase [28]. Accordingly, potential NANDA diagnoses within this pattern include *Ineffective Coping* and *Readiness for Enhanced Coping*. The practical implication for nursing is the design of interventions that guide patients from cycles of avoidance and denial toward stress-management skills, peer and family support, safe therapeutic communication, and follow-up aimed at preventing treatment discontinuation.

### Value–belief pattern

The findings showed that some participants achieved meaning-making and emotional comfort through religious and spiritual beliefs [29]. In Iranian society, religion can serve as an important source of hope and resilience; however, when accompanied by stigmatizing interpretations, it may also reinforce shame and self-blame [30]. Therefore, care approaches should be culturally sensitive and nonjudgmental. Diagnoses related to spiritual well-being enable nurses, with the patient’s consent, to use spiritual resources to strengthen hope without allowing these resources to become instruments of blame [31].

In conclusion, this study demonstrates that perceived stigma profoundly affects multiple dimensions of health among people living with HIV. Utilizing NANDA-based nursing diagnosis patterns provides a systematic approach for understanding these experiences and translating them into effective, person-centered, and culturally responsive nursing interventions. Integrating these diagnostic frameworks into routine HIV care may enhance continuity of care, strengthen professional practice, and ultimately improve the quality of life of affected individuals.

Despite providing in-depth and meaningful findings, this study has several limitations that should be considered when interpreting the results. Due to the qualitative design and relatively small sample size, the findings are not intended to be statistically generalized to all people living with HIV but rather to provide in-depth insight into participants’ lived experiences. The reliance on self-reported data may have resulted in underreporting of sensitive experiences, particularly regarding stigma, sexuality, and family conflict, due to fear of judgment, social consequences, or breaches of confidentiality. In addition, the sensitive nature of HIV and its strong social stigma in Iranian society may have discouraged some participants from fully disclosing their experiences, potentially concealing deeper psychological distress, risky behaviors, or unmet care needs. Limited access to highly marginalized individuals and those disengaged from healthcare services may have led to the underrepresentation of more severe or complex experiences. Therefore, the perspectives of individuals who are completely outside formal care systems may not be fully reflected in the present findings. Furthermore, another important limitation of this study is the gender imbalance in the sample, with a predominance of male participants. Although this partly reflects the gender distribution of registered HIV cases in Iran, it may also be influenced by sociocultural factors. Women living with HIV often experience higher levels of perceived stigma, moral judgment [32], and fear of social consequences, which may reduce their willingness to disclose their status and participate in interviews [33]. As a result, some women may have avoided discussing their experiences or declined participation altogether. This may have led to the underrepresentation of female perspectives and gender-specific challenges, particularly in relation to reproductive health, motherhood, intimate relationships, and gender-related stigma. Therefore, caution is needed when generalizing these findings to female populations. Future studies using larger, more diverse, and gender-balanced samples, as well as mixed-method approaches, are recommended to further validate and expand these findings and to better capture women’s experiences and care needs.

## Conclusion

The findings of this study indicate that perceived stigma plays a central role in shaping and exacerbating the care needs of people living with HIV, affecting multiple dimensions of their physical, psychological, social, sexual, and spiritual health. Directed content analysis of the data within the framework of predefined NANDA nursing diagnosis patterns revealed that the experience of stigma can alter patients’ care and adaptation processes through weakened health management, disrupted self-concept, impaired interpersonal and family relationships, sexual and marital challenges, ineffective coping strategies, and redefinition of life meaning.

The use of the NANDA nursing diagnosis framework allowed for the systematic, comprehensive, and practical identification and explanation of human responses to perceived stigma. The results highlight that focusing solely on the clinical aspects of HIV, without addressing the psychosocial and spiritual consequences of stigma, may lead to overlooking an essential component of patients’ needs.

Accordingly, designing and implementing person-centered, family-centered, and culturally sensitive nursing interventions that emphasize stigma reduction, promotion of self-care, enhancement of psychosocial support, improvement of family relationships, and attention to patients’ spiritual needs appears to be essential. Conscious use of NANDA nursing language by nurses can play a critical role in planning comprehensive care, increasing treatment adherence, and improving the quality of life of people living with HIV.

## Supplementary Information

Below is the link to the electronic supplementary material.

Supplementary Material 1

## Data Availability

On request, the corresponding authors will provide the datasets that were used and analyzed in the current study.
