# Supplementary material for: Extraction of needs based on perceived stigma and their alignment with NANDA in people living with HIV in Iran
Source: BMC Nurs. 2026 Mar 25;25:420. doi: 10.1186/s12912-026-04458-6 (PMC13137621; doi:10.1186/s12912-026-04458-6)
Supplement: Supplementary file 1 — Supplementary Material 1 [file 12912_2026_4458_MOESM1_ESM.docx]

**Semi-Structured Interview Guide**

1. Please describe your experience of living with HIV.
2. How do you perceive the stigma associated with HIV?
3. Since your diagnosis, have you felt labeled or socially rejected? Please explain.
4. How have these experiences affected your emotions, self-attitude, and daily life?
5. How has perceived stigma influenced your health care, visits to medical centers, and adherence to treatment?
6. How have these experiences affected your family and social relationships?
7. Has the stigma of the disease impacted your romantic or sexual relationships?
8. What strategies have you used to cope with these conditions and reduce the effects of stigma?
9. What types of support (family, social, spiritual, or professional) have been helpful or could have been helpful for you?
10. In your opinion, how can nurses and the healthcare team better address the needs of people living with HIV
